# Supplementary material for: The Persistence of Cross-Reactive Immunity to Influenza B/Yamagata Neuraminidase Despite the Disappearance of the Lineage: Structural and Serological Evidence
Source: Int J Mol Sci. 2025 Aug 2;26(15):7476. doi: 10.3390/ijms26157476 (PMC12347243; doi:10.3390/ijms26157476)
Supplement: Supplementary file 1 [file ijms-26-07476-s001.zip › ijms-3770859-supplementary.pdf]

**Supplementary data**

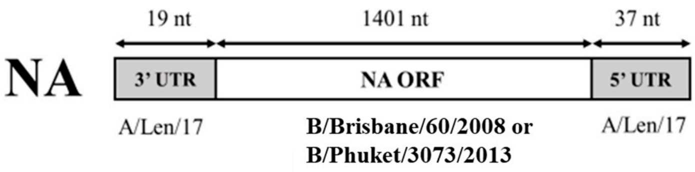


**Figure S1.** Design of the chimeric NA gene. The segment encoding NA is a genetically engineered construct: UTR - untranslated regions, corresponding to fragments of the 6th genetic segment of the A/Leningrad/134/17/57 strain. NA ORF - the coding part of the neuraminidase gene of B/Brisbane/60/2008 (B/Victoria lineage) or B/Phuket/3073/2013 (B/Yamagata lineage).

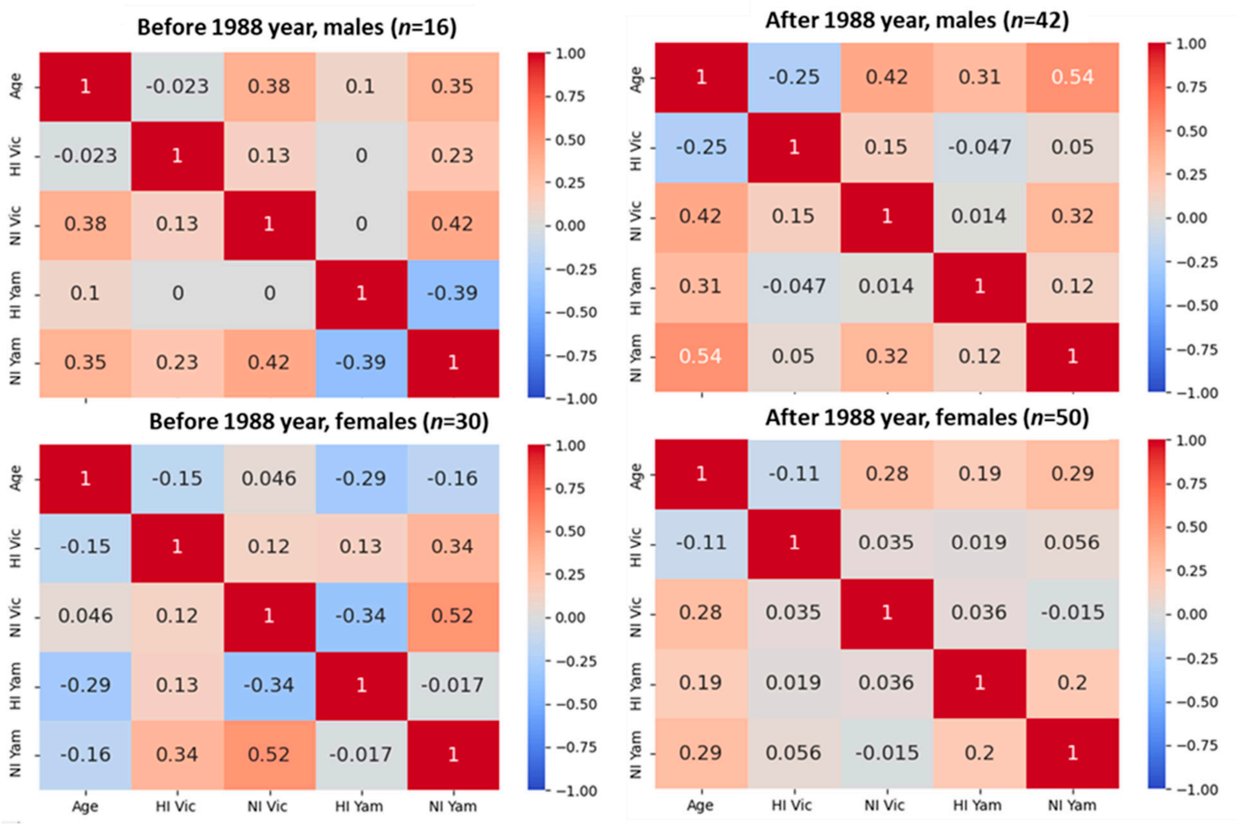


**Figure S2.** Correlation analysis of antibody titers with age in women and men. The data were normalized using the mean normalization method (Z-normalization). The feature intensity pattern was obtained using the built-in functions of the Seaborn library in Python 3. Cells contain the values of the Spearman correlation coefficient ($r_s$). The level of the correlation was determined as follows: $r_s$<0.3– low correlation, $r_s$=0.3-0.49 – moderate correlation, $r_s$=0.5-0.69 – noticeable correlation, $r_s$ ≥0.7 – high correlation.

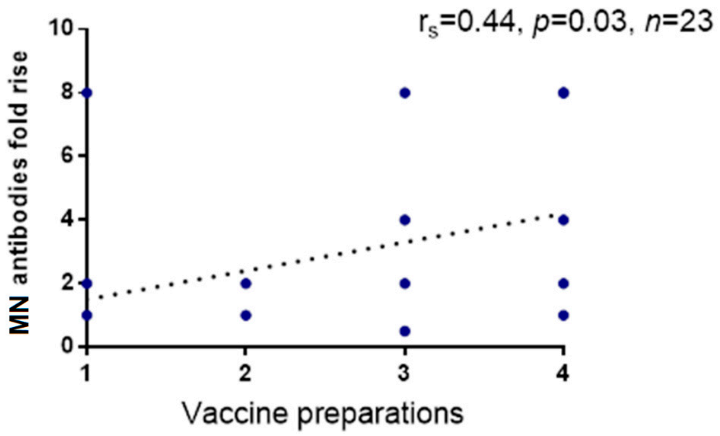


**Figure S3.** Correlation analysis of the MN antibody fold increases to the B/Colorado/06/2017 (B/Victoria) in 23 paired serum samples and influenza vaccine preparations. The influenza vaccines were labeled as follows: 1 – LAIV, 2 – Grippol, 3 – Sovigripp, 4- Ultrix.
